# Supplementary material for: Heparin-based hydrogel scaffolding alters the transcriptomic profile and increases the chemoresistance of MDA-MB-231 triple-negative breast cancer cells
Source: Biomater Sci. 2020 Feb 13;8(10):2786–96. doi: 10.1039/c9bm01481k (PMC7497406; doi:10.1039/c9bm01481k)
Supplement: Supplementary file 2 [file BM-008-C9BM01481K-s002.zip › Supplementary File 4/HP-BhydrogelvControl/Pathways/my_analysis.Gsea.1545201079907/HALLMARK_ALLOGRAFT_REJECTION.html]

Details for gene set HALLMARK\_ALLOGRAFT\_REJECTION[GSEA]

|  || Dataset | expr.class.cls#HYD\_versus\_CONTROL.class.cls#HYD\_versus\_CONTROL\_repos |
| Phenotype | class.cls#HYD\_versus\_CONTROL\_repos |
| Upregulated in class | CONTROL |
| GeneSet | HALLMARK\_ALLOGRAFT\_REJECTION |
| Enrichment Score (ES) | -0.29113778 |
| Normalized Enrichment Score (NES) | -1.3236384 |
| Nominal p-value | 0.024193548 |
| FDR q-value | 0.068591915 |
| FWER p-Value | 0.427 |
Table: GSEA Results Summary

  

Fig 1: Enrichment plot: HALLMARK\_ALLOGRAFT\_REJECTION      
 Profile of the Running ES Score & Positions of GeneSet Members on the Rank Ordered List

  

| PROBE | DESCRIPTION (from dataset) | GENE SYMBOL | GENE\_TITLE | RANK IN GENE LIST | RANK METRIC SCORE | RUNNING ES | CORE ENRICHMENT || 1 | IGSF6 | na |  |  | 302 | 2.668 | 0.0186 | No |
| 2 | PRKCG | na |  |  | 372 | 2.534 | 0.0476 | No |
| 3 | PTPN6 | na |  |  | 544 | 2.288 | 0.0682 | No |
| 4 | TRAF2 | na |  |  | 729 | 2.091 | 0.0855 | No |
| 5 | IL11 | na |  |  | 946 | 1.912 | 0.0988 | No |
| 6 | AKT1 | na |  |  | 1297 | 1.691 | 0.1023 | No |
| 7 | IKBKB | na |  |  | 1605 | 1.528 | 0.1059 | No |
| 8 | ITGB2 | na |  |  | 2483 | 1.179 | 0.0752 | No |
| 9 | HLA-DOB | na |  |  | 2511 | 1.168 | 0.0888 | No |
| 10 | IRF7 | na |  |  | 3003 | 1.036 | 0.0764 | No |
| 11 | IL27RA | na |  |  | 4630 | 0.689 | 0.0001 | No |
| 12 | ICOSLG | na |  |  | 4783 | 0.662 | 0.0007 | No |
| 13 | LIF | na |  |  | 5094 | 0.614 | -0.0076 | No |
| 14 | CSK | na |  |  | 5268 | 0.590 | -0.0091 | No |
| 15 | EGFR | na |  |  | 5500 | 0.552 | -0.0141 | No |
| 16 | CD8A | na |  |  | 5633 | 0.534 | -0.0141 | No |
| 17 | TLR6 | na |  |  | 5743 | 0.520 | -0.0131 | No |
| 18 | INHBB | na |  |  | 6568 | 0.407 | -0.0511 | No |
| 19 | NLRP3 | na |  |  | 6756 | 0.386 | -0.0559 | No |
| 20 | IL18 | na |  |  | 7027 | 0.356 | -0.0654 | No |
| 21 | IL6 | na |  |  | 7244 | 0.333 | -0.0725 | No |
| 22 | TNF | na |  |  | 7258 | 0.331 | -0.0689 | No |
| 23 | LTB | na |  |  | 7357 | 0.318 | -0.0699 | No |
| 24 | JAK2 | na |  |  | 7561 | 0.298 | -0.0767 | No |
| 25 | EIF3A | na |  |  | 7731 | 0.284 | -0.0819 | No |
| 26 | HLA-G | na |  |  | 7771 | 0.277 | -0.0804 | No |
| 27 | HLA-DQA1 | na |  |  | 8229 | 0.229 | -0.1014 | No |
| 28 | IL7 | na |  |  | 8303 | 0.219 | -0.1024 | No |
| 29 | PSMB10 | na |  |  | 8309 | 0.219 | -0.0998 | No |
| 30 | IL12A | na |  |  | 8449 | 0.206 | -0.1044 | No |
| 31 | RPS9 | na |  |  | 8818 | 0.164 | -0.1216 | No |
| 32 | ACVR2A | na |  |  | 8850 | 0.159 | -0.1212 | No |
| 33 | EIF4G3 | na |  |  | 9120 | 0.137 | -0.1335 | No |
| 34 | IL1B | na |  |  | 9136 | 0.136 | -0.1325 | No |
| 35 | IL4R | na |  |  | 9138 | 0.135 | -0.1309 | No |
| 36 | HLA-DMB | na |  |  | 9253 | 0.123 | -0.1352 | No |
| 37 | SOCS1 | na |  |  | 9324 | 0.113 | -0.1374 | No |
| 38 | CCND2 | na |  |  | 9609 | 0.088 | -0.1512 | No |
| 39 | EIF3D | na |  |  | 9654 | 0.079 | -0.1525 | No |
| 40 | AARS | na |  |  | 9792 | 0.065 | -0.1588 | No |
| 41 | C2 | na |  |  | 9829 | 0.060 | -0.1599 | No |
| 42 | TGFB1 | na |  |  | 9937 | 0.048 | -0.1649 | No |
| 43 | ICAM1 | na |  |  | 9949 | 0.046 | -0.1649 | No |
| 44 | DYRK3 | na |  |  | 10124 | 0.025 | -0.1737 | No |
| 45 | BCL3 | na |  |  | 10566 | -0.012 | -0.1966 | No |
| 46 | TAP1 | na |  |  | 10646 | -0.022 | -0.2005 | No |
| 47 | ELF4 | na |  |  | 11360 | -0.098 | -0.2366 | No |
| 48 | HLA-DMA | na |  |  | 11401 | -0.101 | -0.2374 | No |
| 49 | CCND3 | na |  |  | 11490 | -0.109 | -0.2406 | No |
| 50 | NME1 | na |  |  | 11542 | -0.117 | -0.2418 | No |
| 51 | TAPBP | na |  |  | 11603 | -0.126 | -0.2433 | No |
| 52 | CTSS | na |  |  | 11932 | -0.167 | -0.2583 | No |
| 53 | HLA-E | na |  |  | 12076 | -0.183 | -0.2634 | No |
| 54 | BRCA1 | na |  |  | 12249 | -0.206 | -0.2698 | No |
| 55 | ETS1 | na |  |  | 12310 | -0.215 | -0.2702 | No |
| 56 | CCL2 | na |  |  | 12375 | -0.222 | -0.2707 | No |
| 57 | HLA-DOA | na |  |  | 12515 | -0.226 | -0.2750 | No |
| 58 | IFNAR2 | na |  |  | 12530 | -0.229 | -0.2728 | No |
| 59 | TLR1 | na |  |  | 12783 | -0.255 | -0.2827 | No |
| 60 | TLR3 | na |  |  | 12803 | -0.258 | -0.2804 | No |
| 61 | FAS | na |  |  | 12828 | -0.261 | -0.2783 | No |
| 62 | TLR2 | na |  |  | 12836 | -0.262 | -0.2753 | No |
| 63 | HDAC9 | na |  |  | 12887 | -0.270 | -0.2744 | No |
| 64 | CD74 | na |  |  | 12971 | -0.280 | -0.2751 | No |
| 65 | LY75 | na |  |  | 13081 | -0.296 | -0.2770 | No |
| 66 | RPS19 | na |  |  | 13137 | -0.305 | -0.2760 | No |
| 67 | APBB1 | na |  |  | 13265 | -0.318 | -0.2785 | No |
| 68 | MTIF2 | na |  |  | 13297 | -0.325 | -0.2760 | No |
| 69 | CD40 | na |  |  | 13309 | -0.327 | -0.2723 | No |
| 70 | INHBA | na |  |  | 13523 | -0.357 | -0.2789 | No |
| 71 | ACHE | na |  |  | 13587 | -0.367 | -0.2775 | No |
| 72 | LYN | na |  |  | 13668 | -0.380 | -0.2768 | No |
| 73 | CFP | na |  |  | 13712 | -0.387 | -0.2740 | No |
| 74 | RARS | na |  |  | 13794 | -0.402 | -0.2731 | No |
| 75 | UBE2N | na |  |  | 14014 | -0.419 | -0.2792 | No |
| 76 | ABCE1 | na |  |  | 14186 | -0.439 | -0.2825 | No |
| 77 | HLA-DRA | na |  |  | 14240 | -0.450 | -0.2794 | No |
| 78 | GBP2 | na |  |  | 14345 | -0.470 | -0.2788 | No |
| 79 | TAP2 | na |  |  | 14396 | -0.476 | -0.2753 | No |
| 80 | ABI1 | na |  |  | 14438 | -0.481 | -0.2713 | No |
| 81 | TGFB2 | na |  |  | 14509 | -0.492 | -0.2686 | No |
| 82 | TIMP1 | na |  |  | 14560 | -0.499 | -0.2648 | No |
| 83 | MRPL3 | na |  |  | 14592 | -0.504 | -0.2599 | No |
| 84 | BCAT1 | na |  |  | 15072 | -0.574 | -0.2776 | No |
| 85 | IL15 | na |  |  | 15098 | -0.579 | -0.2714 | No |
| 86 | CRTAM | na |  |  | 15475 | -0.645 | -0.2828 | Yes |
| 87 | CAPG | na |  |  | 15558 | -0.662 | -0.2786 | Yes |
| 88 | MAP3K7 | na |  |  | 15614 | -0.671 | -0.2728 | Yes |
| 89 | IFNGR2 | na |  |  | 15618 | -0.672 | -0.2643 | Yes |
| 90 | SOCS5 | na |  |  | 15777 | -0.698 | -0.2636 | Yes |
| 91 | GCNT1 | na |  |  | 15821 | -0.708 | -0.2567 | Yes |
| 92 | DARS | na |  |  | 15889 | -0.721 | -0.2509 | Yes |
| 93 | EIF5A | na |  |  | 16278 | -0.806 | -0.2609 | Yes |
| 94 | ST8SIA4 | na |  |  | 16410 | -0.834 | -0.2570 | Yes |
| 95 | WARS | na |  |  | 16504 | -0.855 | -0.2508 | Yes |
| 96 | GLMN | na |  |  | 16516 | -0.858 | -0.2403 | Yes |
| 97 | HCLS1 | na |  |  | 16552 | -0.866 | -0.2310 | Yes |
| 98 | RIPK2 | na |  |  | 16616 | -0.878 | -0.2230 | Yes |
| 99 | BCL10 | na |  |  | 16727 | -0.907 | -0.2170 | Yes |
| 100 | STAT1 | na |  |  | 16812 | -0.929 | -0.2095 | Yes |
| 101 | EREG | na |  |  | 17124 | -1.024 | -0.2126 | Yes |
| 102 | EIF3J | na |  |  | 17206 | -1.053 | -0.2032 | Yes |
| 103 | RPL39 | na |  |  | 17486 | -1.149 | -0.2030 | Yes |
| 104 | TPD52 | na |  |  | 17682 | -1.234 | -0.1973 | Yes |
| 105 | RPL9 | na |  |  | 17783 | -1.280 | -0.1861 | Yes |
| 106 | GALNT1 | na |  |  | 18057 | -1.420 | -0.1821 | Yes |
| 107 | F2R | na |  |  | 18280 | -1.550 | -0.1737 | Yes |
| 108 | RPS3A | na |  |  | 18308 | -1.570 | -0.1549 | Yes |
| 109 | HIF1A | na |  |  | 18348 | -1.603 | -0.1363 | Yes |
| 110 | SRGN | na |  |  | 18448 | -1.670 | -0.1199 | Yes |
| 111 | IFNGR1 | na |  |  | 18491 | -1.701 | -0.1002 | Yes |
| 112 | DEGS1 | na |  |  | 18558 | -1.750 | -0.0811 | Yes |
| 113 | NCK1 | na |  |  | 18624 | -1.812 | -0.0611 | Yes |
| 114 | CD47 | na |  |  | 18753 | -1.956 | -0.0426 | Yes |
| 115 | UBE2D1 | na |  |  | 19044 | -2.500 | -0.0256 | Yes |
| 116 | B2M | na |  |  | 19069 | -2.604 | 0.0068 | Yes |
Table: GSEA details [plain text format]

  

Fig 2: HALLMARK\_ALLOGRAFT\_REJECTION      
 Blue-Pink O' Gram in the Space of the Analyzed GeneSet

  

Fig 3: HALLMARK\_ALLOGRAFT\_REJECTION: Random ES distribution      
 Gene set null distribution of ES for **HALLMARK\_ALLOGRAFT\_REJECTION**
